# A nomogram improves AJCC stages for colorectal cancers by introducing CEA, modified lymph node ratio and negative lymph node count

**DOI:** 10.1038/srep39028

**Published:** 2016-12-12

**Authors:** Zhen-yu Zhang, Wei Gao, Qi-feng Luo, Xiao-wei Yin, Shiva Basnet, Zhen-ling Dai, Hai-yan Ge

**Affiliations:** 1 Department of Gastrointestinal Surgery, Shanghai East Hospital, Tongji University School of Medicine, Shanghai, China; 2 Department of General Surgery, Qingpu Branch of Zhongshan Hospital, Fudan University, Shanghai, China

## Abstract

Lymph node stages (pN stages) are primary contributors to survival heterogeneity of the 7^th^ AJCC staging system for colorectal cancer (CRC), indicating spaces for modifications. To implement the modifications, we selected eligible CRC patients from the Surveillance Epidemiology and End Results (SEER) database as participants in a training (*n* = 6675) and a test cohort (*n* = 6760), and verified tumor deposits to be metastatic lymph nodes to derive modified lymph node count (mLNC), lymph node ratio (mLNR), and positive lymph node count (mPLNC). After multivariate Cox regression analyses with forward stepwise elimination of the mLNC and mPLNC for the training cohort, a nomogram was constructed to predict overall survival (OS) via incorporating preoperative carcinoembryonic antigen, pT stages, negative lymph node count, mLNR and metastasis. Internal validations of the nomogram showed concordance indexes (c-index) of 0.750 (95% CI, 0.736–0.764) and 0.749 before and after corrections for overfitting. Serial performance evaluations indicated that the nomogram outperformed the AJCC stages (c-index = 0.725) with increased accuracy, net benefits, risk assessment ability, but comparable complexity and clinical validity. All the results were reproducible in the test cohort. In summary, the proposed nomogram may serve as an alternative to the AJCC stages. However, validations with longer follow-up periods are required.

Colorectal cancer (CRC) is the third leading cause of cancer morbidity and mortality worldwide^1^. Despite its increasing complexity, the American Joint Committee on Cancer (AJCC) staging manual, which classifies tumors from the perspective of pathological anatomy, remains the cornerstone for the treatment and prognosis of patients with CRC^2^. The latest (7th edition) AJCC staging manual for CRC specifies metastases in the lymph nodes and tumor deposits (TDs) as the primary evidence of advanced disease, which results in a significant difference in the selection of subsequent adjuvant therapies and in the prediction of patient outcomes. However, survival heterogeneity is frequently present when patients with the same AJCC stages encounter distinct outcomes^3^. This is particularly prominent in patients with stage II and stage III CRC^3^. The current strategy, which determines nodal stages by positive lymph node count (PLNC) and TDs, may constitute the basis of the pN stages (pathological node stages) as a major contributor to the observed survival heterogeneity when the AJCC staging system is used.

The pN stages have several limitations, which indicates that this classification can be modified. First, the pN stages do not consider the effect of TDs on survival when lymph node metastases are present^2^, although the TDs have a similar prognostic impact as metastatic lymph nodes^4^,^5^. Second, the precision of the classification by PLNC depends on adequate assessments of the lymph node count (LNC), yet the LNC tends to be confounded by many operator, patient and tumor factors^6^,^7^. Consequently, the prognostic significance of the LNC is inconsistent among published observational studies^6^,^7^. No widely accepted minimum requirement of LNC is available to determine whether the identified PLNCs are adequate to maintain the accuracy of node staging^7^. Third, other node-related parameters such as negative lymph node count^8^,^9^ (NLNC) and lymph node ratio^6^,^9^,^10^ (LNR) have been demonstrated to be associated with the survival of patients with CRC, while neither the NLNC or LNR is incorporated into the AJCC staging system. Fourth, the pN stages categorize the positive lymph node count (PLNC) and are unable to incorporate continuous variables, which leads to additional loss of information and predictive accuracy. Lastly, the introduction of biomarkers such as the preoperative expression of carcinoembryonic antigen (CEA) may offer extra precision in the prediction of CRC disease status, which might help to ease increased concerns regarding the anatomical basis of the pN stages^11^.

In the present study, we anticipated that the expression of CEA, the presence of TDs and node-related parameters including the LNC, NLNC, PLNC and LNR could explain and address to a certain degree the survival heterogeneity caused by the pN stages. Modifications of the pN stages by the addition of CEA expression, TDs and node-related factors to a multivariate nomogram might improve its predictive accuracy for CRC. To test this hypothesis, we retrospectively reviewed relevant clinical-pathological variables and the vital status of CRC patients from the Surveillance Epidemiology and End Results (SEER) database. The first aim of this study was to verify the basis for modifying the pN stages by the above-mentioned parameters and to show whether TDs might be incorporated as metastatic lymph nodes. The second aim was to determine and validate the optimal multivariate model that was used to establish the predictive nomogram after the modifications. This study may help us understand the survival heterogeneity complicated by the pN stages and may offer patients with CRC an improved prognostic tool without increased complexity.

## Methods

### Patients and eligibility criteria

The SEER program (http://seer.cancer.gov) is maintained by the National Cancer Institute and is a national database of cancer statistics in the United States^12^. The data on cancer research are freely available to the public upon submission of a signed data-use agreement (http://seer.cancer.gov/data/sample-dua.html) to the SEER administration^12^. The experimental protocols used in our study were exempt from review by the ethics committee of the Shanghai East hospital since the data were anonymously extracted and analyzed. Informed consents from participants were also waived due to the complete anonymity of the patients. The study was conducted according to the TRIPOD statement^13^ and adhered to the Declaration of Helsinki for medical research involving human subjects^14^.

In the present study, any CRC patients from the SEER database who were diagnosed in 2010 and 2011 were considered for inclusion in a training cohort and a test cohort, respectively. However, patients were excluded if they met the following criteria: (1) were not diagnosed with adenocarcinoma, (2) unproven diagnosis by surgical pathology, (3) history of malignancy, (4) multiple primary tumors, (5) preoperative/intraoperative radiation therapy, (6) unknown or borderline CEA status, (7) pTis lesions or inconsistent/insufficient information to specify the tumor-node-metastasis (TNM) stages, (8) unknown number of TDs or unknown LNC or PLNC, (9) follow-ups with incomplete dates or follow-ups of less than one month and (10) inactive follow-ups or unknown outcomes.

### Variables and endpoint

The variables that were evaluated were as follows: sex, age, race, tumor location, grade, perineural invasion, CEA expression, TDs, LNC, PLNC, NLNC, LNR, the 7th AJCC/TNM stages, postoperative radiation, survival (in months) and vital status. Among them, the NLNC and the LNR were derived from both the LNC and the PLNC. The endpoint we used was overall survival (OS), which was determined by the vital status.

### Statistical analyses

Discontinuous variables were presented as frequencies while continuous variables were presented as medians and ranges due to skewed distributions. Cumulative survival rates among patients with different pN stages with and without stratifications were plotted using the Kaplan-Meier (K-M) curve method and were compared by log-rank test. To modify the pN stages, each TD was quantified as a metastatic lymph node and the node-parameters were recalculated accordingly to yield the modified LNC (mLNC), PLNC (mPLNC), LNR (mLNR) and AJCC (mAJCC) stages. Based on the training cohort, the mLNC, mPLNC, mLNR, NLNC, CEA expression, pT stages and M stages were then incorporated into a multivariate Cox regression analysis with a forward stepwise elimination of relatively unimportant variables. Advantages of the final multivariate model were attested by comparisons with the AJCC and mAJCC stages using goodness of fit (log-likelihood), Akaike information criterion (AIC) and concordance index (c-index). Next, the nomogram was constructed based on the final model of the training cohort. The performance of the nomogram was internally evaluated by c-index, 200-resample bootstrap validation, calibration and the area under the time-dependent receiver operating characteristic (ROC) curve (AUC) at different time points. External validation was achieved by applying the nomogram to the test cohort using similar statistics. Decision curve analysis^15^ (DCA) was also performed to compare the threshold probabilities and the net benefits associated with the nomogram and the AJCC stages. Lastly, to demonstrate the ability of the nomogram to make risk assessments, each patient in the training cohort was given a total score based on the nomogram. Risk classifications at the overall stage level were illustrated with K-M curves after the patients were divided into different prognostic groups according to percentile scores. Risk stratifications for individual AJCC stages as well as for patients who received postoperative radiation were performed using similar methods. All the analyses were processed by the SPSS 18.0 (SPSS Inc., Chicago, IL, USA) and R 3.2.3 programs. By convention, only a two-sided *P* value < 0.05 was considered statistically significant.

## Results

### Characteristics of the study cohorts

**Figure 1:**
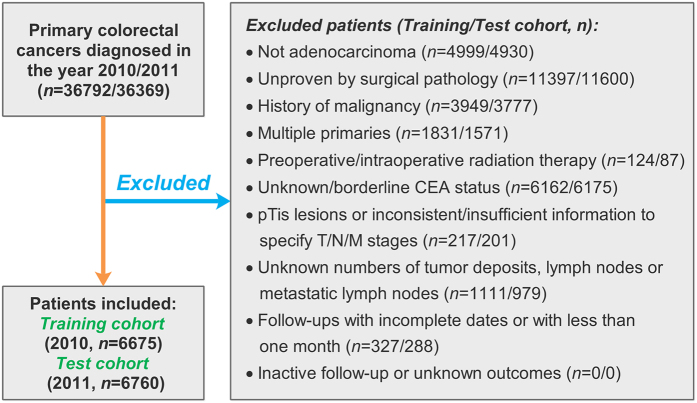
Flow chart of study development.

Although 36,792 and 36,369 patients were identified separately, 6675 and 6760 patients met the eligible criteria for the training and test cohorts, respectively (Fig. 1). Descriptive characteristics and the variables assessed in the two cohorts are shown in Tables 1 and 2.

### Evaluation of the pN stages

**Figure 2:**
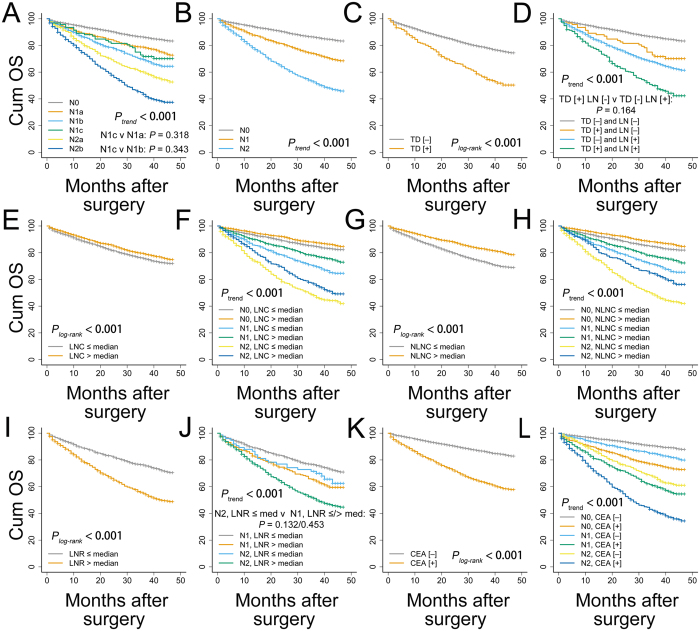
Evaluations on the pN stages with Kaplan-Meier curve analyses. (**A**) pN stages, (**B**) broad node stages, (**C**) TD, (**D**) stratification by TD status, (**E**) LNC, (**F**) stratification by LNC, (**G**) NLNC, (**H**) stratification by NLNC, (**I**) LNR, (**J**) stratification by LNR, (**K**) CEA, (**L**) stratification by CEA. The median LNC and NLNC were 17 and 16, respectively. The analyses for LNR were based on patients with lymph node metastasis (*n* = 2796) with a median LNR of 0.15. All log-rank *P* values for trend and pairwise comparisons were <0.05 unless otherwise specified. TD, tumor deposit; LNC, lymph node count; NLNC, negative lymph node count; LNR, lymph node ratio; CEA, carcinoembryonic antigen; Cum OS, cumulative overall survival.

The results of the K-M curve analyses for the training cohort (Fig. 2) showed that the TDs, LNC, NLNC, LNR and expression of CEA were significantly associated with OS (all *P*_log-rank_ < 0.001). All of these could be used to stratify the pN stages (all *P*_log-rank_ for trend <0.001), while pairwise comparisons revealed some discrepancies among these parameters. For instance, no apparent survival difference was identified between pN1c stage patients and pN1a stage patients (*P*_log-rank_ = 0.318) or between pN1c stage patients and pN1b stage patients (*P*_log-rank_ = 0.343) (Fig. 2A). This was also the case for patients who were TD (−) LN (+) (namely, TD-negative and node-positive cases) and patients who were TD (+) LN (−) (Fig. 2D, *P*_log-rank_ = 0.164). The results insinuated that metastasis in TDs and lymph nodes might have a comparable impact on OS. Furthermore, the survival of node-positive patients was significantly different depending on the TD status (Fig. 2D, *P*_log-rank_ < 0.001), which indicated that the effect of TD could not be ignored when lymph node metastases were present. Moreover, in patients with lymph node metastases (LNR > 0, *n* = 2796), the OS of pN2 stage patients with a decreased LNR (≤median) was comparable to that of pN1 stage patients with an LNR either above or below the median (0.15) (Fig. 2J, *P*_log-rank_ = 0.132 and 0.453). In addition, the expression of CEA exerted a reverse effect on the pN stages (Fig. 2L) as the survival of CEA (−) pN1 patients was better than that of CEA (+) pN0 patients (*P*_log-rank_ < 0.001); moreover, a similar relationship was found between CEA (−) pN2 patients and CEA (+) pN1 patients (*P*_log-rank_ = 0.021). The results for the LNR and CEA expression implied that advanced pN stages were not necessarily associated with a shortened OS. Modifications of the heterogeneous pN stages might bring improved precision to survival estimations.

### Modifications of the N factor

Considering that TDs had a prognostic effect similar to that of positive lymph nodes, these were combined as the mPLNC, the method of which is described above. The results of multivariate Cox analyses for the training cohort are shown in Table 3. The mLNC and mPLNC were excluded due to lack of significance (both *P* = 0.675). Comparisons of the models showed that the multivariate model was the optimal model since it yielded the highest log-likelihood (−13,334.16/−13,421.76/−13,422.92), c-index (0.750/0.725/0.725) and the lowest AIC value (26,686.32/26,859.52/26,861.84) compared with the mAJCC and AJCC stages. The mAJCC stages were also improved with higher goodness of fit and lower information loss compared with the AJCC stages.

### Predictive nomogram

**Figure 3:**
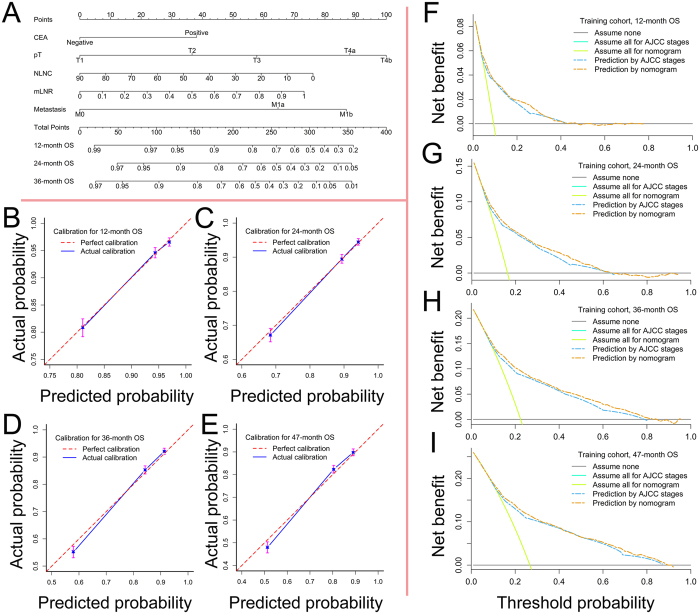
Establishment and internal validation of the nomogram. (**A**) Nomogram based on the training cohort, (**B**) calibration for 12-month OS, (**C**) calibration for 24-month OS, (**D**) calibration for 36-month OS, (**E**) calibration for 47-month OS, (**F**) decision curve analysis for 12-month OS, (**G**) decision curve analysis for 24-month OS, (**H**) decision curve analysis for 36-month OS, (**I**) decision curve analysis for 47-month OS. In the plots of decision curve analysis, the “assume none” lines represented the assumption that no event occurred; while the “assume all” lines represented the assumption that events occurred in all the patients. CEA, carcinoembryonic antigen; pT, pT stages; NLNC, negative lymph node count; mLNR, modified lymph node ratio; OS, overall survival; AJCC, the American Joint Committee on Cancer.

The nomogram was constructed based on the final multivariate model for the training cohort (Fig. 3A).

### Internal and external validations

The c-indexes of the nomogram in the training and test cohorts were 0.750 (95% CI, 0.736–0.764) and 0.770 (95% CI, 0.754–0.786), respectively. Similarly, the bias-corrected c-indexes for the training and test cohorts were 0.749 and 0.769, respectively, which indicates no significant changes. Calibration plots displayed a good agreement between the observed and the nomogram-predicted OS at different time points in both the training (Fig. 3B to E) and test cohorts (see Supplementary Fig. S1). The time-dependent AUCs at 12, 24, 36 and 46 months in the training cohort were 0.754 (95% CI, 0.732–0.776), 0.771 (95% CI, 0.755–0.787), 0.781 (95% CI, 0.767–0.795) and 0.771 (95% CI, 0.747–0.796), respectively; the time-dependent AUCs at 12, 24, and 34 months in the test cohort were 0.780 (95% CI, 0.759–0.801), 0.792 (95% CI, 0.776–0.807) and 0.802 (95% CI, 0.777–0.827), respectively. Additional DCA plots (Fig. 3F to I) showed that the nomogram consistently outperformed the AJCC stages, as the nomogram was associated with improved net benefits (higher lines of prediction by the nomogram). However, the nomogram gave comparable threshold probabilities between which a predictive model was clinically valid. The results of the DCA remained stable in the test cohorts (see Supplementary Fig. S2).

### Risk classifications and stratifications

**Figure 4:**
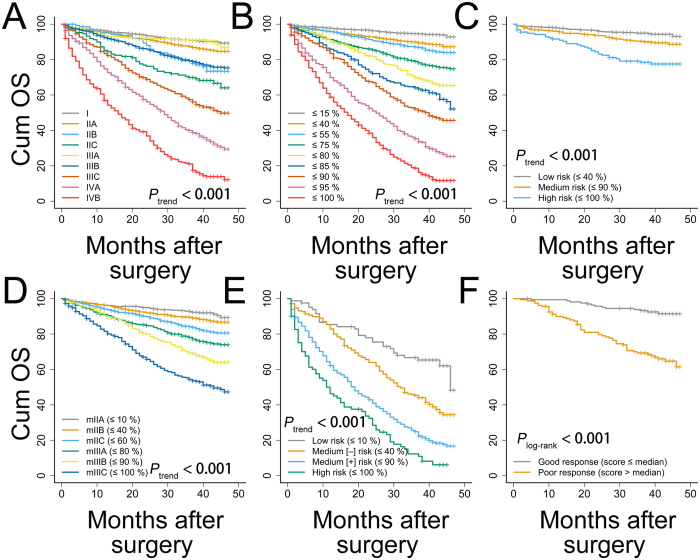
Risk assessments using the nomogram. (**A**) AJCC stages, (**B**) nomogram classifications for stage I–IV, (**C**) nomogram stratifications for stage I, (**D**) nomogram stratifications for stage II–III with modified sub-stages, (**E**) nomogram stratifications for stage IV, (**F**) nomogram stratifications for patients with postoperative radiation (*n* = 325, median score = 155.9). All log-rank *P* values for trend and pairwise comparisons were <0.001 unless otherwise specified. Cum OS, cumulative overall survival.

After the patients were scored and ranked according to percentiles, risk classifications and stratifications were implemented to illustrate the ability of the nomogram to make risk assessments in the training cohort. In general, the nine AJCC stages were unable to accurately predict the OS of patients with CRC, particularly for those with stage II and stage III disease (Fig. 4A, IIIA vs. I, *P*_log-rank_ = 0.766; IIIA vs. IIA, *P*_log-rank_ = 0.080; IIIB vs. IIB, *P*_log-rank_ = 0.776). Conversely, the nomogram was able to classify patients with stage I-IV disease into nine significant prognostic groups (Fig. 4B, all *P*_log-rank_ < 0.016 for pairwise comparisons). Based on the percentile scores of the particular stages, the nomogram could also stratify patients with stage I (Fig. 4C, all *P*_log-rank_ < 0.002 for pairwise comparisons), stage II–III (Fig. 4D, all *P*_log-rank_ < 0.047 for pairwise comparisons) and stage IV (Fig. 4E, all *P*_log-rank_ < 0.001 for pairwise comparisons) disease into a number of significant risk subgroups. Additionally, responses to postoperative radiation therapy (first course therapy) in patients who receive postoperative radiation (*n* = 325) might also be predicted by the nomogram (Fig. 4F, *P*_log-rank_ < 0.001).

## Discussion

In the present study, we evaluated the survival heterogeneity that results from use of the pN stages and proposed a new prognostic nomogram that was able to avoid the limitations associated with the AJCC staging system. The nomogram achieved stable improvements in predictive accuracy, net benefits and reproducibility through the incorporation of the expression of CEA, pT stages, NLNC, mLNR and metastasis without a significant increase in degrees of freedom (*df* = 9).

As supported by a number of previous studies^5^,^16^,^17^,^18^,^19^, there are some reasons that TDs should be considered metastatic lymph nodes irrespective of lymph node status. Most importantly, our study showed that TDs and metastatic lymph nodes had a comparable impact on the survival of patients with CRC and that TDs also imposed risks on node-positive CRC. Consistent with our study, an investigation^16^ of patients with node-positive CRC reported an increased recurrence rate (49.2% vs. 14.4%, *P* < 0.001) and decreased OS (*P* < 0.001) after surgery in those with TDs compared with those without TDs. Other important, supportive reasons include the finding that metastases in the TDs and lymph nodes shared similar recurrence patterns^17^ and that pathologists experience substantial difficulty in the complete differentiation of these two entities^18^,^19^. Actually, our study showed that the mAJCC stages, which were simplified by the combination of TDs and metastatic lymph nodes, achieved a higher log-likelihood and a lower AIC in comparison with conventional AJCC staging. Some studies have also reported that this combination enhanced the diagnostic objectivity^19^ and predictive accuracy^19^,^20^,^21^ of the pN stages.

Due to the aforementioned limitations, the higher pN stages did not seem to necessarily be associated with shortened survival. The results of the K-M curve analyses revealed that use of the pN stages led to both underestimates (i.e., pN2, LNR ≤ median and pN1, CEA (−)) and overestimates in OS (i.e., pN0, CEA (+) and pN1, CEA (+)) of patients with CRC. We observed that 76.5% (224/293) of the stage IIIA patients and 46.0% (684/1490) of the stage IIIB patients in the training cohort constituted 22.2% and 67.7%, respectively, of the pN1 CEA (−) patients (*n* = 1010) who were identified to be at risk for underestimation by the pN stages. We also observed that 17.6% (275/1563) of the stage I patients and 34.1% (609/1787) of the stage IIA patients accounted for 24.3% and 53.8%, respectively, of the pN0 CEA (+) patients (*n* = 1132) whose survival was likely to be overestimated. This explains precisely why some of the stage II patients exhibited a worse survival than stage III patients. In addition, the results indicate that pN1 CEA (−) and pN0 CEA (+) patients may be treated as high-risk stage II patients for whom adjuvant therapies are appropriate, but this requires further validation.

The nomogram successfully avoided the above-mentioned limitations of the pN stages by the inclusion of other node parameters. It was not accidental that the NLNC and mLNR, rather than the mLNC and mPLNC, were prioritized by the multivariate Cox analyses. The mLNR contained additional information about the NLNC, which improved the predictive accuracy of the mPLNC. A recent systematic review confirmed that the prognostic value of the LNR was superior to that of the PLNC^22^. Moreover, the nomogram allowed the mLNR to be continuously represented. This further avoided the problem of the threshold variability in the LNR, which made studies incomparable and hindered the application of the LNR^22^. In contrast to the mLNR, the NLNC and LNC were applicable to patients with either early or advanced CRC. Consistent with many other studies^3^,^8^,^9^,^23^,^24^, our analyses revealed a positive association among the NLNC, LNC and OS. The mechanisms of this association are increasingly linked to confounders that simultaneously correlate with the LNC and survival of patients with CRC^6^,^7^. An emerging role of the adaptive immune response to tumors is also highlighted to characterize the LNC as a patient-specific marker rather than as a quality indicator^25^. Despite the association, the NLNC showed an advantage over the LNC as a more significant predictor in our study. One reason may be that the favorable effect of the NLNC on OS is more relevant and stable than that of the LNC because the LNC in node-positive patients considers the NLNC and PLNC, while the effect of the LNC may be neutralized since the two components exert opposite effects on prognosis. This is in accord with a recent population-based study, in which the 12-node benchmark proved to be an independent predictor of CRC in patients with stage I-III disease (*n* = 13,941, HR = 0.67) but not in patients with stage III-IV disease (*n* = 6810, *P* = 0.136)^24^. Another possible reason is that the influence of the LNC on patient survival is more easily diminished by improvements in the quality of external pathology with increasing awareness of the 12-node minimum requirement^26^. In contrast, the NLNC may be more intrinsically related to enhanced regional lymphocytic reactions that result in an increased NLNC and prolonged survival^27^. Therefore, the NLNC is a better predictor of survival than the LNC.

Together with previous findings, our study provided one of the first nomograms that incorporates CRC patients with and without metastasis using population-based data. This nomogram is also the first CRC prognostic nomogram that contains a modification of the algorithm for the presence of TDs and the pN stages through the incorporation of both the NLNC and the mLNR. Compared with the published nomograms^28^ and the AJCC stages for CRC, our nomogram exhibited improved accuracy without a significant increase in model complexity. Nonetheless, our study does have some limitations that deserve attention. Since the analyses were performed retrospectively, selection biases might be underestimated. The duration of the follow-up periods in both cohorts is relatively short because the SEER program did not collect data on TDs until the year 2010. Although we have demonstrated that the performance of the nomogram is reliable and reproducible, this nomogram may still require validation by independent studies with a longer follow-up period. It should also be noted that the SEER research database lacks chemotherapy information albeit the data are irrelevant to the development of the nomogram. Additionally, the inclusion of new biomarkers such as cell-free DNAs^29^ and circulating tumor cells^30^ may improve the performance of the nomogram. Lastly, tumor location (i.e., right-sided vs. left-sided location) is associated with site-specific genetic alterations^31^ that may biologically determine tumor recurrence and outcome^32^. Thus it may be a simple, reproducible and robust predictor of future modifications of nomograms^33^ and AJCC stages.

In summary, our study demonstrates substantial survival heterogeneity among the pN stages, which decreases the performance of the AJCC staging system. The quantification of TDs as metastatic lymph nodes is an effective and practical modification that improves predictive accuracy. Based on that modification, the nomogram that incorporates CEA expression, pT stages, the NLNC, the mLNR and metastasis has been internally and externally validated as a useful tool for risk assessments. This nomogram also outperformed the conventional AJCC staging system in both the training and test cohorts with increased predictive accuracy and net benefits but with comparable complexity and clinical validity. Thus, this nomogram holds promise for future application in clinical practice. However, this nomogram still requires independent validations with longer durations of follow-up.

## Additional Information

**How to cite this article**: Zhang, Z.-y. *et al*. A nomogram improves AJCC stages for colorectal cancers by introducing CEA, modified lymph node ratio and negative lymph node count. *Sci. Rep.* **6**, 39028; doi: 10.1038/srep39028 (2016).

**Publisher's note:** Springer Nature remains neutral with regard to jurisdictional claims in published maps and institutional affiliations.

## Supplementary Material

Supplementary Information

## Figures and Tables

**Table 1:** Descriptive characteristics of the eligible patients.

Variables	Training cohort (N = 6675)		Test cohort (N = 6760)	
	Frequency	Percent (%)	Frequency	Percent (%)
Sex				
Female	3360	50.3	3337	49.4
Male	3315	49.7	3423	50.6
Age, median, range	65	18–108	66	17–100
Race				
White	5236	78.5	5215	77.1
Black	789	11.8	840	12.4
American Indian/Alaska Native	49	0.7	42	0.6
Asian or Pacific Islander	576	8.6	629	9.3
Unknown	25	0.4	34	0.5
Location				
Colon	5544	83.1	5658	83.7
Rectum	1131	16.9	1102	16.3
Grade				
G1+G2	5279	79.1	5540	82.0
G3+G4	1262	18.9	1108	16.4
Unknown	134	2.0	112	1.6
Perineural Invasion				
Negative	5394	80.8	5695	84.2
Positive	589	8.8	651	9.6
Unknown	692	10.4	414	6.1
Radiation				
None	6296	94.3	6388	94.5
After surgery	325	4.9	300	4.4
Refused or Unknown	54	0.8	72	1.1
Follow-up, month	39	1–47	28	1–35
Number of events, OS	1636	24.5	1227	18.2
12-month OS, %	90.7	—	91.0	—
24-month OS, %	83.7	—	84.2	—
36-month OS, %	77.5	—	78.9^a^	—
47-month OS, %	72.8	—	—	—

OS, overall survival. ^a^Cumulative survival rate at 35 months in the test cohort.

**Table 2:** Assessed variables of the eligible patients.

Variables	Training cohort (N = 6675)		Test cohort (N = 6760)	
	Frequency	Percent (%)	Frequency	Percent (%)
CEA				
Negative	4127	61.8	4184	61.9
Positive	2548	38.2	2576	38.1
TD, median, range	0	0–74	0	0–81
LNC, median, range	17	1–90	17	1–90
PLNC, median, range	0	0–46	0	0–52
NLNC, median, range	16	0–90	16	0–90
LNR, median, range	0	0–1	0	0–1
pT stage				
T1	818	12.3	802	11.9
T2	1101	16.5	1147	17.0
T3	3798	56.9	3766	55.7
T4a	561	8.4	663	9.8
T4b	397	5.9	382	5.6
pN stage				
N0	3786	56.7	3883	57.4
N1a	846	12.7	844	12.5
N1b	884	13.2	925	13.7
N1c	93	1.4	96	1.4
N2a	591	8.9	549	8.1
N2b	475	7.1	463	6.9
Metastasis				
M0	5852	87.7	5906	87.4
M1a	564	8.4	579	8.6
M1b	259	3.9	275	4.0
AJCC stage				
I	1563	23.4	1584	23.4
IIA	1787	26.8	1825	27.0
IIB	144	2.2	175	2.6
IIC	149	2.2	124	1.8
IIIA	293	4.4	303	4.5
IIIB	1490	22.3	1465	21.7
IIIC	426	6.4	430	6.4
IVA	564	8.4	579	8.6
IVB	259	3.9	275	4.0

TD, tumor deposit; LNC, lymph node count; PLNC, positive lymph node count; NLNC, negative lymph node count; LNR, lymph node ratio; AJCC stage, the 7th American Joint Committee on Cancer stage.

**Table 3:** Multivariate Cox regression analysis in the training cohort.

Covariates	HR	95% CI	*P* value
CEA	1.767	1.585–1.969	<0.001
pT stage (ref = pT1)			
pT2	1.746	1.288–2.367	<0.001
pT3	2.399	1.832–3.141	<0.001
pT4a	3.774	2.816–5.059	<0.001
pT4b	4.496	3.333–6.066	<0.001
NLNC (every node increase)	0.987	0.981–0.994	<0.001
mLNR (every percent increase)	1.011	1.008–1.013	<0.001
Metastasis (ref = M0)			
M1a	2.620	2.295–2.991	<0.001
M1b	3.619	3.061–4.279	<0.001
mLNC	—	—	0.675
mPLNC	—	—	0.675

HR, hazard ratio; 95% CI, 95% confident interval; ref, referent; CEA, carcinoembryonic antigen; NLNC, negative lymph node count; mLNR, modified lymph node ratio; mLNC, modified lymph node count; mPLNC, modified positive lymph node count.
