# Supplementary material for: A nomogram improves AJCC stages for colorectal cancers by introducing CEA, modified lymph node ratio and negative lymph node count
Source: Sci Rep. 2016 Dec 12;6:39028. doi: 10.1038/srep39028 (PMC5150581; doi:10.1038/srep39028)
Supplement: Supplementary Information [file srep39028-s1.pdf]

Supplementary Information

A nomogram improves AJCC stages for colorectal cancers by introducing CEA, modified lymph node ratio and negative lymph node count

Zhen-yu Zhang, Wei Gao, Qi-feng Luo, Xiao-wei Yin, Shiva Basnet, Zhen-ling Dai, Hai-yan Ge*

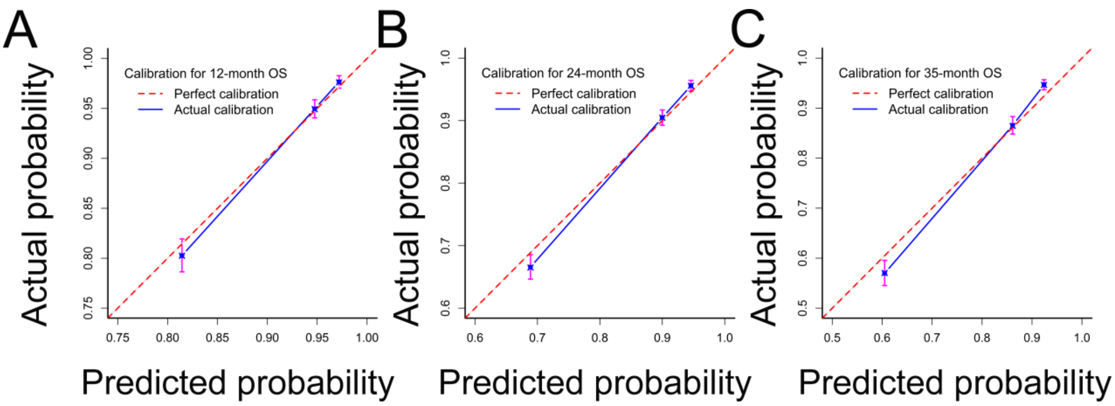

Figure S1. Calibration plots in the test cohort. (A) Calibration for 12-month OS, (B) calibration for 24-month OS, (C) calibration for 35-month OS. OS, overall survival.

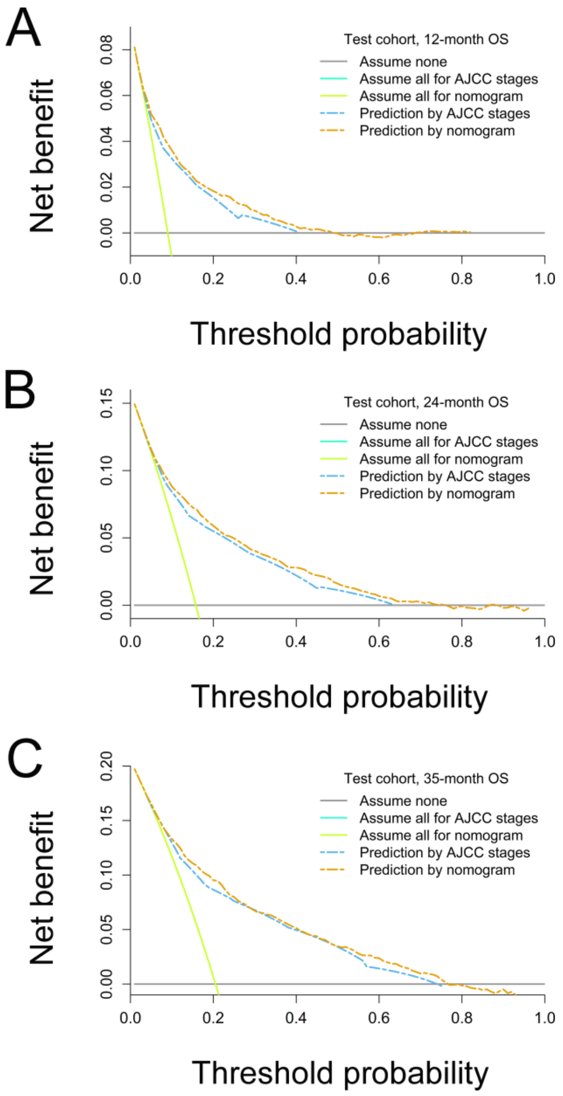

Figure S2. Decision curve analyses in the test cohort. (A) 12-month OS, (B) 24-month OS, (C) 35-month OS. In the plots, the "assume none" lines represented the assumption that no event occurred; while the "assume all" lines represented the assumption that events occurred in all patients. OS, overall survival.
